# Nitrogen addition increases sexual reproduction and improves seedling growth in the perennial rhizomatous grass *Leymus chinensis*

**DOI:** 10.1186/s12870-020-2307-8

**Published:** 2020-03-06

**Authors:** Song Gao, Junfeng Wang, Johannes M. H. Knops, Jiao Wang

**Affiliations:** 1 Graduate School, Changchun University, Changchun, 130022 China; 2 Key Laboratory of Vegetation Ecology, Ministry of Education, Institute of Grassland Science, School of Life Sciences, Northeast Normal University, Changchun, 130024 China; 3 Department of Health and Environmental Sciences, Xi’an Jiaotong Liverpool University, Suzhou, 215123 China

**Keywords:** Nitrogen deposition, Seed production, Seedling growth, Dominant species, Semi-arid grassland

## Abstract

**Background:**

The Eurasian steppe is an important vegetation type characterized by cold, arid and nitrogen poor conditions. At the Eastern edge, including in the Songnen grassland, the vegetation is dominated by *Leymus chinensis* (henceforth *L. chinensis*) and is increasing threatened by elevated anthropogenic nitrogen deposition. *L. chinensis* is a perennial grass that mainly reproduces vegetatively and its sexual reproduction is limited. However, sexual reproduction plays an important role influencing colonization after large disturbances. To develop an understanding of how elevated nitrogen deposition changes the plant community structure and functioning we need a better understanding how sexual reproduction of *L. chinensis* changes with nitrogen enrichment. Here we report on a field experiment where we added 10 g N m^− 2^ yr^− 1^ and examined changes in seed traits, seed germination and early seedling growth.

**Results:**

Nitrogen addition increased seed production by 79%, contributing to this seed increases were a 28% increase in flowering plant density, a 40% increase in seed number per plant and a 11% increase in seed weight. Seed size increased with a 42% increase in large seeds and a 49% decrease in the smallest seed size category. Seed germination success improved by 10% for small seeds and 18% for large seeds. Combined, the increased in seed production and improved seed quality doubled the potential seed germination. Subsequent seedling above and below-ground biomass also significantly increased.

**Conclusions:**

All aspects of *L. chinensis* sexual reproduction increased with nitrogen addition. Thus, *L. chinensis* competitive ability may increase when atmospheric nitrogen deposition increases, which may further reduce overall plant diversity in the low diversity Songnen grasslands.

## Background

In many grasslands, the diversity of the vegetation is driven by a diversity of low abundance broadleaf forbs, whereas most of the biomass is contributed by a low diversity of grasses [1]. In more stressful grasslands, such as the Songnen grasslands, which have a high pH, arid climate and strong nitrogen limitation [2], the overall plant diversity is low and the grass component can be dominated by a single species, such as *L. chinensis* [3]. In such a vegetation the structure and functioning of the entire ecosystem is closely associated with the traits and ecology of the dominant species. In most perennial grasslands worldwide, the dominant grasses mainly reproduce vegetatively and seed production is often low [4]. However, sexual reproduction is still important because seeds are key for long distance dispersal and seeds play an important role to reestablish vegetation after major disturbances [5]. Sexual reproduction is influenced by the seed number and the seed quality [6]. Particularly, larger seeds are beneficial because of increased seedling growth especially under unfavorable conditions [7].

Worldwide, in many grasslands there is increased nitrogen deposition [8, 9] including in China. Background annual atmospheric nitrogen deposition in the North China Plain is in the order of 2 g N m^− 2^ yr^− 1^, whereas current rates are as high as 15 g N m^− 2^ yr^− 1^ at some areas [10]. Similar increases are predicted for many more areas in the North China Plain grasslands. Most studies examining the impact of elevated nitrogen deposition in grasslands focus on net primary productivity [11], or vegetative plant reproduction, and largely ignore changes in plant sexual reproduction. However, increased nitrogen availability also changes plant growth [12] and frequently increases total seed production, increases the relative biomass allocation to reproduction [13, 14], and seed production becomes more stable over time [15–17]. Nitrogen fertilization increases seed production by either increasing seed number, the weight of each seed or a combination of both. However, species differ in this, some species such as *Festuca rubra* L. do not change seed weight with nitrogen addition [18], whereas as other grasses such as *Lolium perenne* L. do increase seed weight [19].

*Leymus chinensis* (Trin.) Tzvel, is the dominant perennial, rhizomatous grass widely distributed in the eastern regions of the Eurasian steppe zone, including the Songnen plain [2]. In Songnen grasslands, it is contributing 80–90% to the total vegetation biomass [3]. Previous nitrogen addition studies have focused on community productivity and diversity and found that nitrogen addition lowers plant diversity and increases net primary productivity because of an increase in shoot density and individual shoot weight [20, 21]. However, how sexual reproduction of *L. chinensis* changes in relation to increased nitrogen has largely been ignored. We simulated atmospheric nitrogen disposition in a nitrogen addition experiment to examine changes in sexual reproduction in *L. chinensis*. We hypothesized that nitrogen addition (1) increases seed production, because of increased resource availability; (2) increases average seed weight, because increased resources allow all seeds to fully develop thereby reduces the amount of inferior seed; (3) improves seed quality as measured by increased seed germination and seedling growth, because of larger seeds.

## Results

**Fig. 1:**
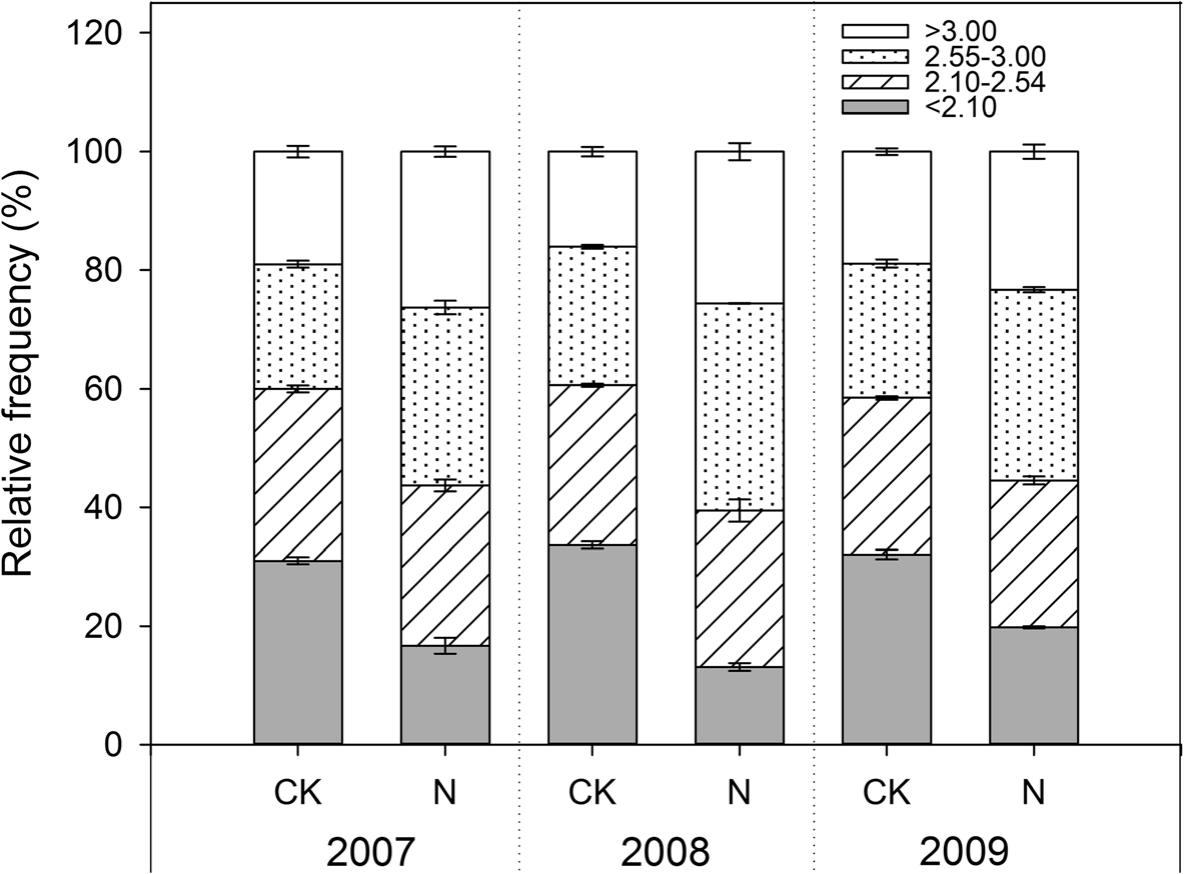
Seed size distribution of four seed weight fractions for control and nitrogen addition treatments for three years. Presented are the means ±1 S. E for 6 replications

Nitrogen addition significantly increased sexual reproduction, specifically the flowering plant density, seed number per plant, thousand seed weight, and the seed number per square meter produced (Tables 1 and 2). Some aspects of sexual reproduction such as the flowering plant number and seed density varied among the years, whereas the seed number per plant and thousand seed weight did not vary among the years (Table 1). Nitrogen addition induced a 48.7% decrease in the small seed (< 2.1 mg) proportion and an average of 42.3% increase in the large seed (2.6–3.0, > 3.0 mg) proportion (Fig. 1).

**Table 1:** Results of the two-way ANOVA examining the effects of the experimental year and nitrogen addition on vegetation, plant and seed changes. Presented are the F and *P* values of ANOVA’s, *n* = 6 for all vegetation and plant measurements and *n* = 3 for all germination measurements

Dependent trait	Year (Y)		Nitrogen addition (N)		Y × N	
	*F*	*P*	*F*	*P*	*F*	*P*
Flowering plant density (no. m^2^)	4.003	**0.029**	51.809	**0.000**	0.084	0.919
Seed no. per plant	1.347	0.276	9.696	**0.004**	0.708	0.501
Thousand seeds weight (g)	3.186	0.057	41.986	**0.000**	0.371	0.693
Seed no. (m^2^)	4.052	**0.028**	34.716	**0.000**	1.394	0.264
Potential germination no. (m^2^)	9.908	**0.001**	50.813	**0.000**	3.419	**0.046**
Germination success (%)						
< 2.1 mg	9.564	**0.003**	0.503	0.492	0.503	0.617
2.1–2.5 mg	2.207	0.153	7.091	**0.021**	2.207	0.153
2.6–3.0 mg	28.123	**0.000**	3.280	**0.005**	0.822	0.463
> 3.0 mg	15.449	**0.000**	31.223	**0.000**	2.092	0.166
Germination rate						
< 2.1 mg	4.193	**0.028**	6.820	**0.023**	1.373	0.290
2.1–2.5 mg	4.332	**0.038**	6.379	**0.027**	0.034	0.967
2.6–3.0 mg	69.209	**0.000**	41.444	**0.000**	4.663	0.032
> 3.0 mg	57.227	**0.000**	62.229	**0.000**	16.346	**0.000**
Seedling shoot biomass (g individual^−1^)						
< 2.1 mg	2.316	0.141	15.235	**0.002**	0.431	0.659
2.1–2.5 mg	1.664	0.230	15.698	**0.002**	0.554	0.589
2.1–2.5 mg	1.112	0.361	5.844	**0.032**	1.165	0.345
> 3.0 mg	6.854	**0.010**	74.894	**0.000**	10.609	**0.002**
Seedling root biomass (g individual^−1^)						
< 2.1 mg	1.804	0.206	8.207	**0.014**	0.294	0.750
2.1–2.5 mg	1.149	0.349	17.894	**0.001**	2.710	0.093
2.1–2.5 mg	1.160	0.345	21.745	**0.001**	0.098	0.907
> 3.0 mg	0.110	0.896	5.558	**0.036**	0.777	0.482

† Bold text represents the significant values was less than 0.05 (*P* < 0.05)

**Table 2:** Flowering plant density and seed number per plant, thousand seed weight, and seed density in control plots and nitrogen addition plots. The data are expressed as means ±1 SE, n = 6 per treatment

	2007		2008		2009	
	Control	Nitrogen	Control	Nitrogen	Control	Nitrogen
Flowering plant density (no.m^2^)	82.2bc ± 3.5	104.2a† ± 5.2	79.7bc ± 2.8	104.0a ± 3.7	72.5c ± 3.6	93.8ab ± 3.9
Seed no. per plant	14.0ab ± 1.2	22.1a ± 2.5	13.5ab ± 3.5	18.1ab ± 8.1	13.1b ± 1.5	16.4ab ± 5.2
Thousand seeds weight (g)	2.1c ± 0.1	2.3ab ± 0.1	2.2bc ± 0.1	2.5a ± 0.1	2.2bc ± 0.1	2.4ab ± 0.1
Seed no. per (m^2^)	1151.0bc ± 42.8	2300.5a ± 299.6	1089.3c ± 68.3	1897.8ab ± 86.5	960.3c ± 34.7	1522.3bc ± 119.0

† For each variable, values with different letters indicate the significant differences between control and nitrogen addition treatment among three experimental years (*P* < 0.05, from post hoc comparison). For ANOVA results, see Table 1

**Fig. 2:**
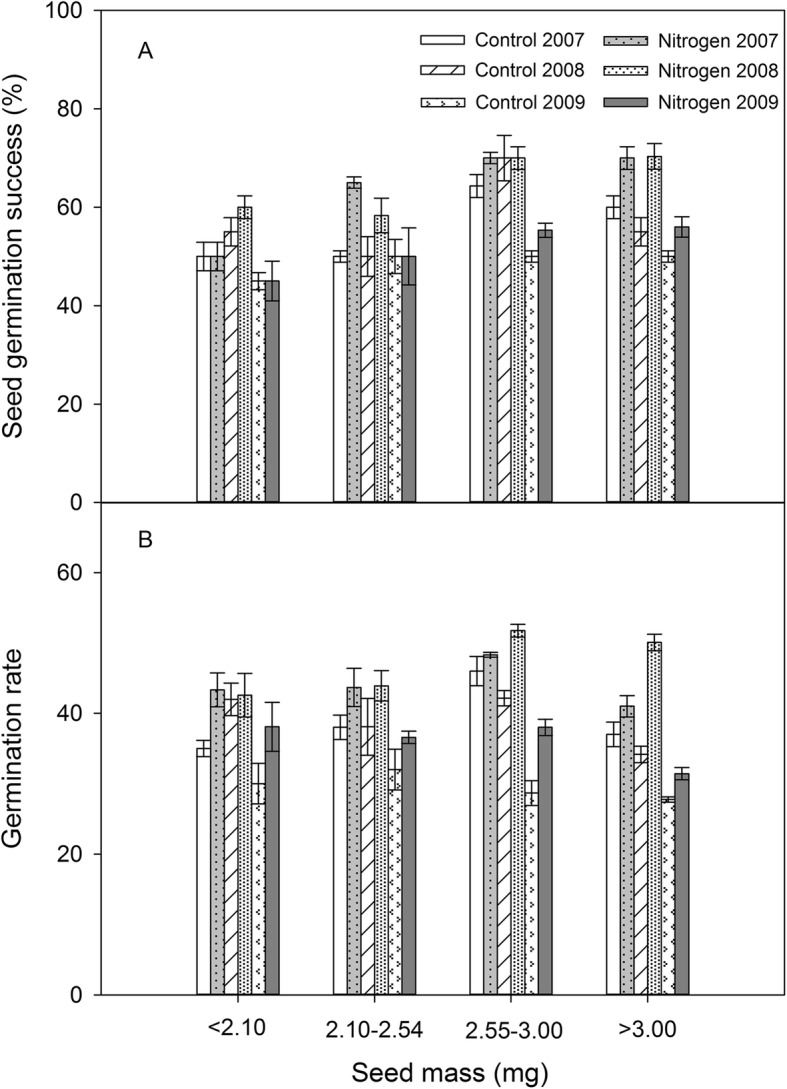
Seed germination (**a**) and seed germination rate (**b**) of four seed weight fractions in three years for control and nitrogen addition treatments. Germination rate was calculated according to the following equation: germination rate = ΣG/t, where G is the percentage of seed germinated at 1 day interval and t is the total germination period. Presented are the means ±1 S. E for 3 replications

Germination success of large and medium seeds increased significantly with nitrogen addition, however there was no change for the smallest seed size category (Fig. 2a, Table 1). Germination success also varied significantly among years, but only for the largest seed category (Fig. 2a, Table 1). In contrast to the germination success, the germination rates of all seed sizes significantly increased with nitrogen addition and varied significantly among the years (Fig. 2b, Table 1).

**Fig. 3:**
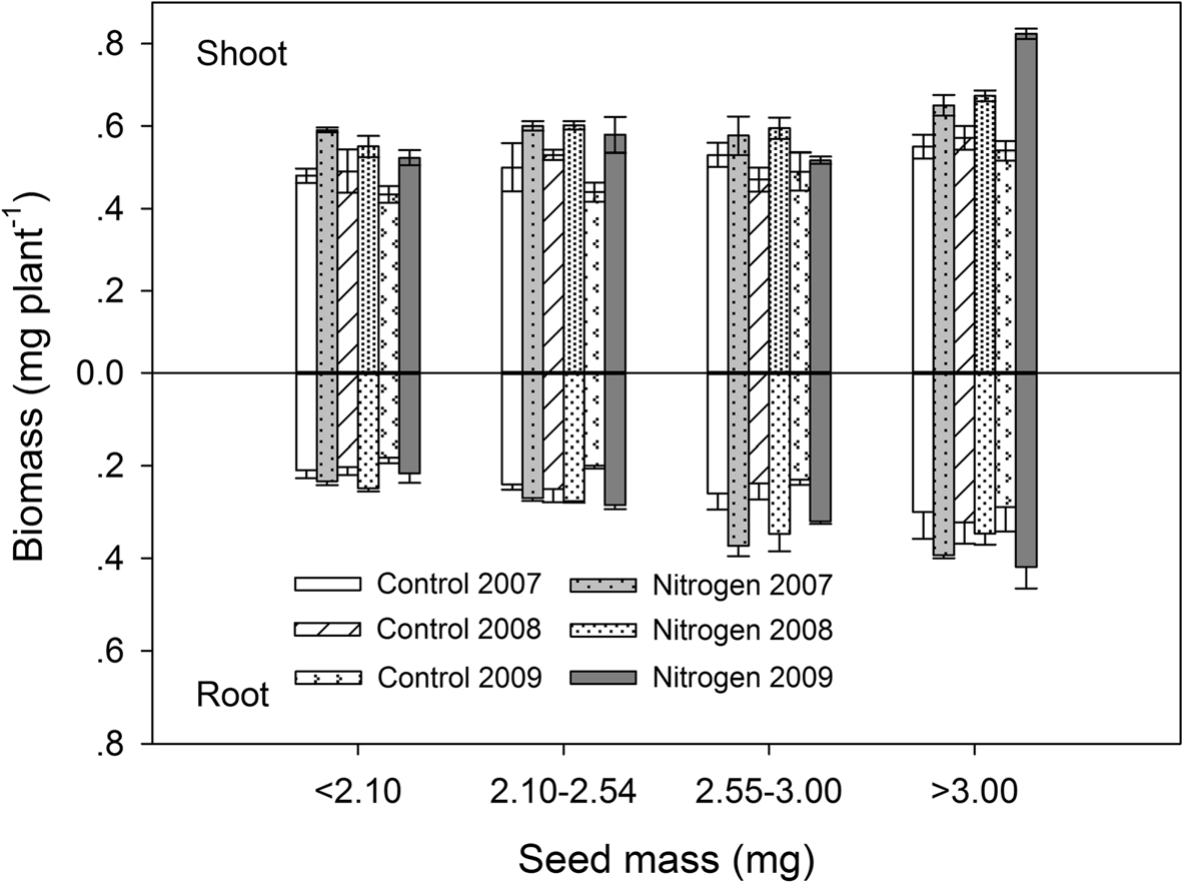
Aboveground shoot and belowground root growth for four seed weight fractions in three years for control and nitrogen addition treatments. Presented are the means ±1 S. E for 3 replications

Seedling growth, as indicated by the seedling shoot and root biomass, in both large and small seeds increased significantly with nitrogen addition consistently among years, except for the largest seed category (Fig. 3).

**Fig. 4:**
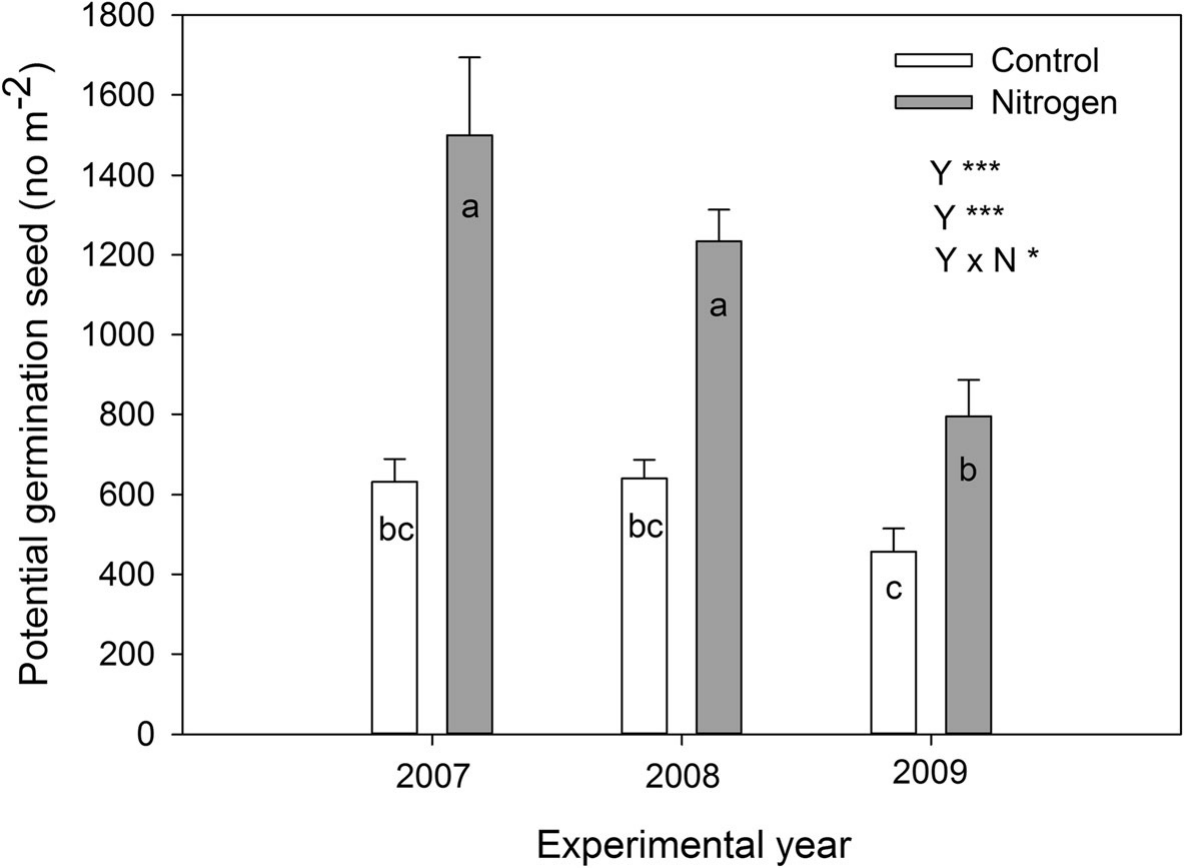
Potential number of germinating seed density based on control versus nitrogen seed production. Presented are the means ±1 S. E for 6 replications. Different letters indicate the significant differences between control and nitrogen addition treatment among three experimental years (*P* < 0.05, from post hoc comparison). For detail ANOVA results, see Table 1

The potential number of seeds that are produced that can germination significantly increased with nitrogen addition, but also showed significant differences among the three sampling years (Table 1). This pattern in potential seeds produced available for germination matched the pattern in the flowering plant density (Fig. 4, Table 2).

## Discussion

In line with the first and second predictions, nitrogen addition increased all sexual reproduction components that contribute to total seed production of *L. chinensis,* i.e. flowering plant density, seed number per plant, and seed weight. The seeds produced on each plant shoot and the seed weight are determined by the growing conditions during the year of flowering, hence nitrogen addition has a direct influence [22]. Increased seed weight is likely caused by the increased resource availability resulting from nitrogen addition [23, 24]. However, an important component leading to increased seed production was the higher flowering plant density. Flowering shoots originate from belowground tillers that were produced in the previous late summer and early fall, thus are influenced by nitrogen addition in the year before seed production. Previous research in tall fescue and ryegrass grassland added nitrogen not every year and shows that both flowering plant density and seed weight did not respond much to nitrogen addition [18, 19]. Thus, because different components that determine total seed production were influenced in different years, multiple years of nitrogen addition may promote responses similar with natural nitrogen deposition in a long-term. Do note that we simulated the increase in nitrogen deposition by adding a onetime nitrogen pulse each year. This differs from current and anticipated future increased atmospheric nitrogen deposition; the wet part of atmospheric deposition is more closely linked to the annual precipitation patterns and the dry deposition component is more evenly distributed throughout the year. It is unclear if our experimental onetime pulse nitrogen addition benefits *L. chinensis* more than other plant species, and there is a need for experiments that more closely use a continuous addition of nitrogen that better mimics natural patterns of atmospheric nitrogen deposition.

**Fig. 5:**
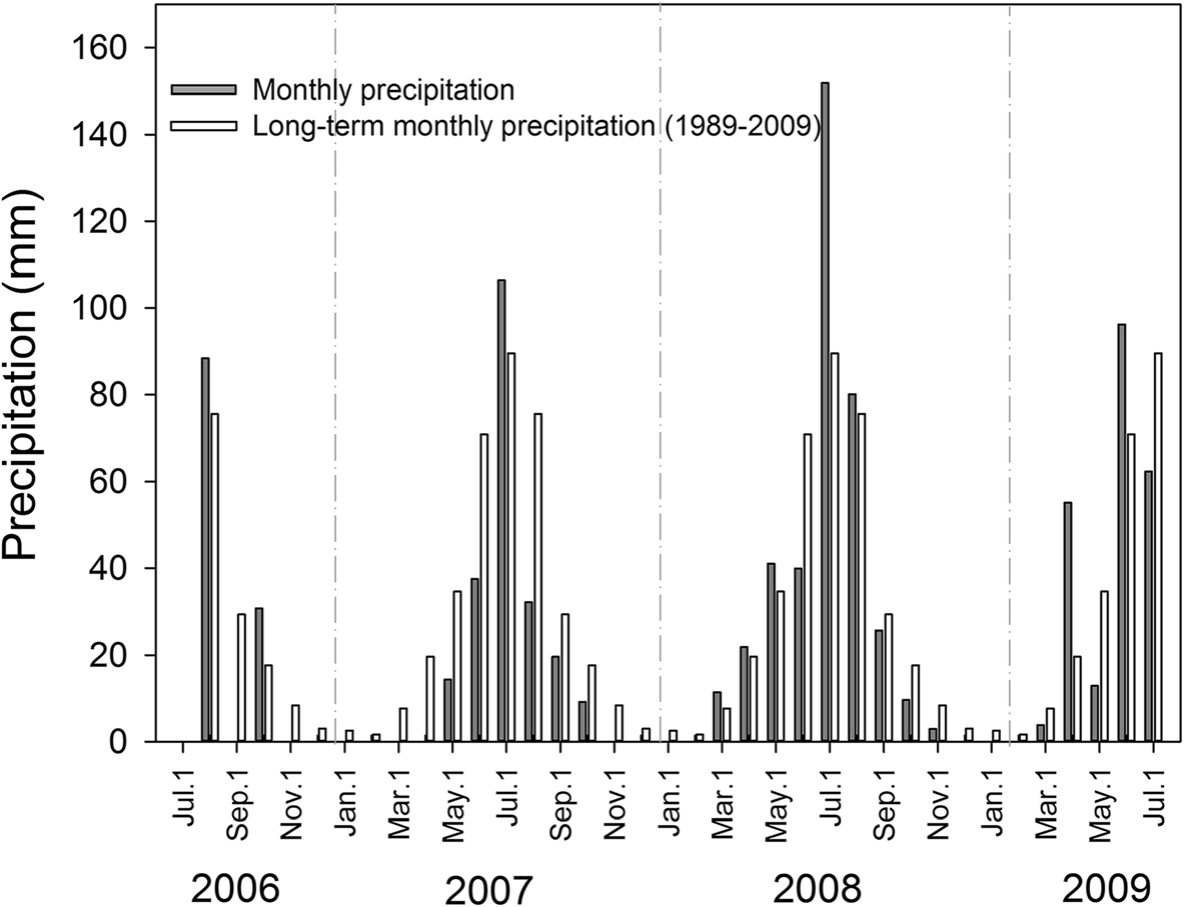
Average and actual monthly precipitation. Long-term average precipitation is based on monthly average precipitation from 1989 to 2009, collected at the weather station of the Ecosystem Field Station of Northeast Normal University, which is located close to the research site. The actual monthly data is from an eddy meteorological tower located adjacent (~ 200 m) to the experimental plots

Nitrogen addition reduced both the number and proportion of small seeds and increased the germination success and germination rate of all larger seed categories, which consistent with the third prediction. Small seeds typically are lower quality seeds with reduced germination and seedling growth rates. Previous research on *L. chinensis* has also shown that small, inferior seeds may contain chemical or microorganisms that inhibits the germination of all, including larger seeds [25]. Regardless of the mechanism, clear is that the increase in absolute and proportional seed weight is the likely main driver of improved total seed quality. Nitrogen addition also improved seedling growth in both small and big seeds, which typically is caused by increased metabolic resources present in the nitrogen induced larger seed [26, 27]. This increase in resources can speed up seed germination because the radicle can break the seed coat faster, speeding up the nutrient absorption from the soil, and hence seedling growth [26–28]. This faster seedling establishment is an advantage for new stand establishment because earlier developing seedlings can obtain more resources, thereby gaining a competitive advantage to later germinating seeds [29, 30]. We also found a significant difference in seed germination success and rate among our three sampling years. Rainfall was above normal during the early part of the growing season when seeds develop in the first 2 years of our experiment (Fig. 5). This increased rainfall may have contributed to this increased germination success and rate, because adequate soil moisture supply is beneficial to seed development [31].

In summary, nitrogen addition not only significantly promotes seed productivity and size, nitrogen addition also increases seed germination and growth. Thus, with anticipated future increases in atmospheric nitrogen deposition, *L. chinensis* competitive ability may benefit from such increased nitrogen availability because of the increased seed number that can increase the soil seed bank and larger seeds that enhance germination and seedling growth. Such an increase in competitive ability may lead to increased dominance of *L. chinensis*, as has been shown in many nitrogen addition studies that show increased dominance of a single grass species [1, 11]*.* The Songnen grasslands are characterized by low plant diversity, and increased *L. chinensis* dominance with increased atmospheric nitrogen addition may further decrease overall plant diversity as has been documented in many other nitrogen addition experiments [32], and studies using atmospheric nitrogen deposition gradients [33].

## Conclusions

We show here that nitrogen addition significantly increases all aspects of sexual reproduction in *L. chinensis*. Nitrogen addition increased flowering plant density and seed number per plant, also increased seed quality, by increasing the average seed weight, increasing the proportion of larger seed and decreasing the smaller seed proportion. This increase in seed quality improved both seed germination and subsequent seedling growth. Thus, under anticipated future increased nitrogen deposition, *Leymus chinensis* competitive dominance may further increase.

## Methods

### Study site

The study site was conducted at the Grassland Ecosystem Field Station of the Northeast Normal University at Songnen Grassland, China (123 °44′E and 44 °40′N, 137.8–144.8 m a.s.l.). The mean annual precipitation of the area is 360 mm (1989–2017), with most of the rainfall in this monsoon climate occurring during the growing season from June to August. The air temperature is highest in July, 22~25 °C and coldest in January − 22~ − 16 °C. The major soil type is Mollisols. The soil contains 19.6 ± 1.3 g nitrogen kg^− 1^, 29.4 ± 3.0 g organic carbon kg^− 1^, and has a pH of 8.64 ± 0.2 in the 0–25 cm depth horizon (*n* = 3 for all measurements [29]. The plant community at the experimental site is dominated by *L. chinensis* with about 80% cover, other common species are *Calamagrostis epigeios, Chloris virgata* and *Puccinellia tenuiflora*. Currently, the atmospheric nitrogen deposition rate at our study location in the Northeast region of China is about 5 g N m^− 2^ yr^− 1^ and is projected to continue to increase [34, 35]. This research examines a native plant species that does not require any special permit. Research permission was obtained for this project from the Key Laboratory of Vegetation Ecology, Ministry of Education, Northeast Normal University.

### Species description

*L. chinensis*, a perennial, rhizomatous grass widely distributed in the eastern regions of the Eurasian steppe zone [30]. *L. chinensis* is tolerant to saline-alkali soil, extreme drought and cold, and has high palatability for livestock likes cow and sheep [31]. *L. chinensis* flowers in late May to early June, seed matures in late July and grows vegetatively until October, after which aboveground shoots die and plants go dormant until the next spring. The flowering and vegetative plant shoots have a height of 70 cm and 50 cm, and each shoot has 3 to 8 leaves.

### Experimental design and measurements

This experiment used a randomized complete block design with six replicate blocks laid out in twelve plots, with each block including one nitrogen addition plot and one control plot. Plot size was 3 × 4 m and plots were separated by a 3 m border. 10 g m^− 2^ of nitrogen was added to simulate nitrogen deposition using ammonium nitrate fertilizer (99% purity) at the end of June each year. Nitrogen addition start in 2006 and we measured the vegetation in 2007 to 2009. In May 2006, a permanent 1 × 1 m quadrat was located in the center of each plot. The density of *L. chinensis* vegetative and flowering (spike) shoot population was determined at the mature seed stage at the end of July in each year. 20 flowering plant shoots in each plot were randomly sampled to count with the spike seed and floret numbers (Voucher specimens deposited in a public samples storage room of Institute of Grassland Science, Northeast Normal University. The formal identification of samples was done by SG and [JW]1). Filled seeds with glumes were air-dried for 3 weeks in laboratory conditions before measuring the thousand-seed weight (seed from the same block was considered as one sample and labelled using sample time with block identification. For details see the supplementary material). The thousand-seed weight was calculated from the weight of 100 seeds multiplied by 10. The seed number per square meter was calculated based on the flowering plants density multiplied by the plant shoot seed number.

Each year five hundred mature seeds in each treatment were randomly selected and we calculated the proportion of seed mass in each class with three replications (the seed was divided into 4 groups with mass of < 2.10, 2.10–2.54, 2.55–3.00, > 3.00 mg). Then, 20 seeds of each weight group were randomly selected with three replications, placed into a sealed Petri dish (11 cm diameter) with double-layer filter paper for the germination test. The Petri dishes were placed into growth chamber (HPG-400, Haerbin DL technology development Co. Ltd., Haer12bin, China) with a 30 °C/14 h light, 20 °C/10 h dark.

Before starting the germination test, seeds were sterilized using 5% potassium permanganate solution for 10 min. The cumulative seed germination rate was recorded each day until no more seeds germinated over a 20-day period. All seedlings were grown for 15 days and then harvested to determine seedling shoot biomass (SSB) and seedling root biomass (SRB). All samples were oven dried at 65 °C for 24 h. Germination success (GS) = *Gt*/*S* × 100, where Gt is the germinated seed number and S is the sum number of germinated seeds and potentially viable seeds [36]. Germination rate (GR) = ∑*G*/*t*, where G is the percentage of germinated seeds per day and *t* is the total germination period. Potential seed germination (the proportion of seeds that will germinate) per square meter was calculated using: (SG) = $\sum \limits_i^4 TS\times RF\times GS$, where TS is the total number of seeds per square meter; RF is the proportion of each mass class and GS is the germination success (%).

### Data analysis

To determine the effect of nitrogen addition effect, yearly differences and year by nitrogen interaction on seed production, germination and seedling growth, we used General Linear Models. Comparisons of means between control and nitrogen addition treatments during experimental period were performed with Tukey’s post hoc test for each variable. All statistical analyses were performed using the SPSS (SPSS 22.0, SPSS Inc., Chicago, USA). As criteria for statistical significance we used α = 0.05.

## Supplementary information

**Additional file 1.**

## Data Availability

The dataset supporting the conclusions of this article is included within the supplementary file (Electronic supplementary material-data.xlsx). It is available from the corresponding author on reasonable request.

## References

[1] Hautier Y, Seabloom E, Borer E, Adler PB, Harpole WS, Hillebrand H, Lind EM, MacDougall AS, Stevens CJ, Bakker JD, Buckley YM, Chu C, Collins SL, Daleo P, Damschen EI, Davies KF, Fay PA, Firn J, Gruner DS, Jin VL, Klein JA, Knops JMH, La Pierre KJ, Li W, McCulley RL, Melbourne BA, Moore JL, O'Halloran LR, Prober SM, Risch AC, Sankaran M, Schuetz M, Hector A (2014). Eutrophication weakens stabilizing effects of diversity in natural grasslands. Nature.

[2] Wang JF, Shi YJ, Ao YN, Yu DF, Wang J, Gao S, Knops JMH, Li ZJ (2019). Summer drought decreases *L.chinensis* productivity through constraining the bud, tiller and shoot production. J Agron Crop Sci.

[3] Zhu TC. Biological ecology of *L. chinensis chinensis*. Jilin Science and Technology Press, Changchun, China (In Chinese) 2004; pp. 24–25.

[4] Dalgleish HJ, Hartnett DC (2006). Below–ground bud banks increase along a precipitation gradient of the north American Great Plains: a test of the meristem limitation hypothesis. New Phytol.

[5] Walck JL, Hidayati SN, Dixon KW, Thompson K, Poschlod P (2011). Climate change and plant regeneration from seed. Glob Chang Biol.

[6] Hovenden MJ, Wills KE, Chaplin R, VanderSchool JK (2008). Warming and elevated CO_2_ affect the relationship between seed mass, germinability and seedling growth in *Austrodanthonia caespitosa*, a dominant Australian grass. Glob Chang Biol.

[7] Breen AN, Richards JH (2008). Irrigation and fertilization effects on seed number, size, germination and seedling growth: implications for desert shrub establishment. Oecologia.

[8] Galloway JN, Townsend AR, Erisman JW, Erisman JW, Bekunda M, Cai ZC, Freney JR, Martinelli LA, Seitzinger SP, Sutton MA (2008). Transformation of the nitrogen cycle: recent trends, questions, and potential solutions. Science.

[9] Sutton MA, Bleeker A (2013). Environmental science: the shape of nitrogen to come. Nature.

[10] He C, Wang X, Liu XJ, Fangmeier A, Christie P, Zhang FS (2010). Nitrogen deposition and its contribution to nutrient inputs to intensively managed agricultural ecosystems. Ecol Appl.

[11] Wang JF, Knops JMH, Brassil CE, Mu CS (2017). Increased productivity in wet years drives a decline in ecosystem stability with nitrogen additions in arid grasslands. Ecology.

[12] Lau J, Peiffer J, Reich P, Tiffin P (2008). Transgenerational effects of global environmental change: long-term CO_2_ and nitrogen treatments influence offspring growth response to elevated CO_2_. Oecologia.

[13] Tilman D (1987). Secondary succession and the pattern of plant dominance along experimental nitrogen gradients. Ecol Monogr.

[14] Gotelli NJ, Ellison AM (2002). Nitrogen deposition and extinction risk in the northern pitcher plant. Ecology.

[15] Callahan HS, Del Fierro K, Patterson AE, Zafar H (2008). Impacts of elevated nitrogen inputs on oak reproductive and seed ecology. Glob Chang Biol.

[16] Kelly D, Sork VL (2002). Mast seeding in perennial plants: why, how, where?. Annu Rev Ecol Syst.

[17] Tanentzap AJ, Lee WG, Coomes DA (2012). Soil nutrient supply modulates temperature-induction cues in mast-seeding grasses. Ecology.

[18] Young William C., Chilcote David O., Youngberg Harold W. (1907). Spring-Applied Nitrogen and Productivity of Cool-Season Grass Seed Crops. Agronomy Journal.

[19] Hebblethwaite PD, Wright D. Noble A. Some physiological aspects of seed yield in *Lolium perenne* L. (perennial ryegrass). In P.D. Hebblethwaite (ed.) seed production. Proc. Easter School in Agric. Sci., 28th, London. September 1978. Butterworth: Univ. of Nottingham; 1980;71–90.

[20] Zhang YH, Feng JC, Isbell F, Lü XT, Han XG (2015). Productivity depends more on the rate than the frequency of N addition in a temperate grassland. Sci Rep.

[21] Yang GJ, Lü XT, Stevens CJ, Zhang GM, Wang HY, Wang ZW, Zhang ZJ, Liu ZY, Han XG (2019). Mowing mitigates the negative impacts of N addition on plant species diversity. Oecologia.

[22] Wang JF, Li XY, Gao S, Li ZL, Mu CS (2013). Impacts of fall nitrogen application on seed production in *Leymus chinensis*, a rhizomatous perennial grass. Agron J.

[23] Marcelis LFM, Heuvelink E, Hofman-Eijer LRB, Bakker JD, Xue LB (2004). Flower and fruit abortion in sweet pepper in relation to source and sink strength. J Exp Bot.

[24] Sakai S, Sakai A (1996). Why is there variation in mean seed size among plants within single populations? Test of the fertilization efficiency hypothesis. Am J Bot.

[25] Ma HY. Mechanism of deep dormancy and germination in *Leymus chinensis* seed. Thesis of Chinese Academy of Sciences. 2008; pp. 41–55.

[26] Moles AT, Westoby M (2004). Seedling survival and seed size: A synthesis of the literature. J Ecol.

[27] Hanley ME, Cordier PK, May O, Kelly CK (2007). Seed size and seedling growth: differential response of Australian and British Fabaceae to nutrient limitation. New Phytol.

[28] HilleRisLambers J, Harpole WS, Schnitzer S, Tilman D, Reich PB (2009). CO_2_, nitrogen, and diversity differentially affect seed production of prairie plants. Ecology.

[29] Gao S, Wang JF, Zhang ZJ, Dong G, Guo JX (2012). Seed production, mass, germination success, and subsequent seedling growth responses to parental warming environment in *Leymus chinensis*. Crop Pasture Sci.

[30] Hou J, Romo JT (1998). Seed weight and germination time affect growth of two shrubs. J Range Manag.

[31] Wang JF, Xie JF, Zhang YT, Gao S, Zhang JT, Mu CS (2010). Methods to improve seed yield of *Leymus chinensis* based on nitrogen application and precipitation analysis. Agron J.

[32] Clark CM, Tilman D (2008). Loss of plant species after chronic low-level nitrogen deposition to prairie grasslands. Nature.

[33] Stevens CJ, Dise NB, Mountford JO, Gowing DJ (2004). Impact of nitrogen deposition on the species richness of grasslands. Science.

[34] Pan YP, Wang YS, Tang GQ, Wu D (2012). Wet and dry deposition of atmospheric nitrogen ate ten sites in northern China. Atmos Chem Phys.

[35] Zhu JX, He NP, Wang QF, Yuan GF, Wen D, Yu GY, Jia YL (2015). The composition, spatial patterns, and influencing factors of atmospheric wet nitrogen deposition in Chinese terrestrial ecosystems. Sci Total Environ.

[36] Lin JX, Shi YJ, Tao S, Yu XY, Yu DF, Yan XF (2017). Seed-germination response of *Leymus chinensis* to cold stratification in a range of temperatures, light and low water potentials under salt and drought stresses. Crop Pasture Sci.

